# Statistical Evidence for the Role of Southwestern Indian Ocean Heat Content in the Indian Summer Monsoon Rainfall

**DOI:** 10.1038/s41598-018-30552-0

**Published:** 2018-08-14

**Authors:** T. Venugopal, M. M. Ali, M. A. Bourassa, Y. Zheng, G. J. Goni, G. R. Foltz, M. Rajeevan

**Affiliations:** 1 Department of Physics, Novosibirsk State University, Novosibirsk, Russia; 2 Department of Meteorology and Oceanography, Andhra University, Visakhapatnam, India; 3 Center for Ocean-Atmospheric Prediction Studies (COAPS), Florida State University, Tallahassee, USA; 4 Indian Institute of Tropical Meteorology, Pune, India; 5 Department of Earth, Ocean and Atmospheric Science, Florida State University, Tallahassee, USA; 6 Physical Oceanography Division, Atlantic Oceanographic and Meteorological Laboratory (AOML)/NOAA, Miami, USA; 7 Ministry of Earth Sciences, Government of India, New Delhi, India

## Abstract

This study examines the benefit of using Ocean Mean Temperature (OMT) to aid in the prediction of the sign of Indian Summer Monsoon Rainfall (ISMR) anomalies. This is a statistical examination, rather than a process study. The thermal energy needed for maintaining and intensifying hurricanes and monsoons comes from the upper ocean, not just from the thin layer represented by sea surface temperature (SST) alone. Here, we show that the southwestern Indian OMT down to the depth of the 26 °C isotherm during January–March is a better qualitative predictor of the ISMR than SST. The success rate in predicting above- or below-average ISMR is 80% for OMT compared to 60% for SST. Other January–March mean climate indices (e.g., NINO3.4, Indian Ocean Dipole Mode Index, El Niño Southern Oscillation Modoki Index) have less predictability (52%, 48%, and 56%, respectively) than OMT percentage deviation (PD) (80%). Thus, OMT PD in the southwestern Indian Ocean provides a better qualitative prediction of ISMR by the end of March and indicates whether the ISMR will be above or below the climatological mean value.

## Introduction

Predicting Indian Summer Monsoon Rainfall (ISMR) is a challenging problem. Although attempts are being made to improve the forecasting skill of the monsoon through numerical and statistical modelling, it remains difficult to predict. One reason for this low skill could be the incorrect representation of the ocean thermal energy that may lead to improper assessments of the influence of the oceans in the prediction models for extreme weather events. ISMR has a substantial impact on the Indian agriculture and economic growth. The Indian monsoon is a coupled phenomenon between ocean, land, and atmosphere. In addition to the atmospheric factors, oceans play a critical role in monsoon physics and prediction. The two main processes that affect ISMR are El Niño/Southern Oscillation^1^ and Indian Ocean Dipole (IOD)^2,3^. Sea Surface Temperature (SST), representing the thin upper layer of the ocean, has been the main oceanographic parameter historically used to infer the influence of IOD and El Niño/Southern Oscillations, although the thermal energy required for the atmospheric processes comes from the upper ocean heat content (OHC). Many times, the thermal energy available in the upper ocean is not reflected in SST. For example, rapid heating and cooling events can quickly erase the thermal signature of subsurface warm or cold features^4^. Smith *et al*.^5^ observed that El Niño forecast models could be improved by initializing with the observed OHC. In addition, OHC has forecasting skill with more lead time as reported by Ji and Leetma^6^ and Latif *et al*.^7^. They found that forecasting skills improved, even with 6–12 months lead times, by initializing the numerical modelling forecast with the OHC. This predictability increases because interannual anomalies in OHC near the equatorial Pacific lead those in equatorial SST by several months^8^. This phase relationship is observed both in ocean models and in observations^9,10^. The atmosphere interacts with the upper ocean rather than with the surface alone, suggesting that ISMR predictions might be improved through the inclusion of upper ocean parameters. In this context, OHC is worthy of consideration for improving ISMR qualitative predictability (meaning the prediction of a weaker or stronger ISMR relative to the climatological mean).

**Figure 1:**
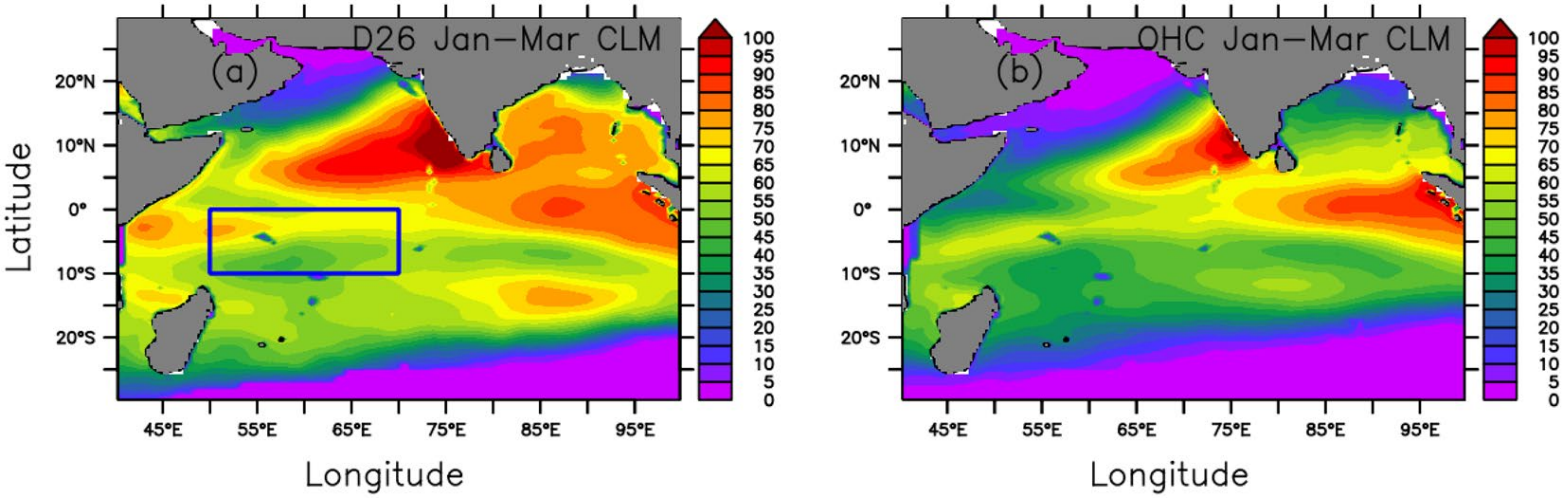
Climatological (1993–2017) spatial distribution of (**a**) D_26_ (in m) and (**b**) OHC (in kJ cm^−2^) during January–March, a transition period between the winter and the southwest monsoon seasons. Rectangular box in (**a**) is the study region of the southwestern Indian Ocean region. This figure was generated using PyFerret V7.1.

In addition to interannual climate variations, OHC is useful for predicting tropical cyclone intensification^11–13^. Ali *et al*.^14,15^ suggested that OHC could be a better parameter than SST in predicting cyclone intensities in the Indian Ocean. Nagamani *et al*.^16^ reported that the OHC from the surface down to the depth of 26 °C isotherm (D_26_) in the Arabian Sea Mini Warm Pool (The region in the southeastern Arabian Sea from 4°–14°N to 68°−78°E) increased during 1998–2010. They speculated that this increase was responsible for the overall decreasing trend in the ISMR during this period. A widely used metric for the upper ocean heat energy is the tropical cyclone heat potential (used here as OHC), the integrated heat energy from the surface to D_26,_ relative to the energy if that layer were uniformly 26 °C, with a minimum value of zero. However, since OHC cannot be integrated into purely atmospheric models, Ali *et al*.^17^ suggested converting OHC to Ocean Mean Temperature (OMT) of the surface to D_26_. They studied the relationships of ISMR with OMT and SST at each 2.5° box in the North Indian Ocean for different months and concluded that OMT in the southwestern Indian Ocean region (50°E–70°E and 10°S–0°N; delimited by the rectangular box in Fig. 1a) has the highest correlation. They also reported that the correlations and confidence levels are greater for OMT compared to SST. They estimated only one correlation coefficient for each 2.5° box for the entire study period of 1993–2013. They did not study the performance of OMT for year-to-year ISMR variations. There appears to be a very non-linear dependency on ocean-related variables, hence a linear correlation for the entire study period (where year-to-year variations are large) is a poor indication of skill in the prediction. Predicting the sign of anomalies is important because it is linked to more/less rainfall, which is critical for agricultural economy of a country, such as India. Herein, we focus on that skill of predicting the ISMR anomaly for different years by considering the same southwestern Indian Ocean region as in Ali *et al*.^17^ to explore the improvements in the predictability of ISMR using OMT of this region in place of SST, in a statistical sense. We further compare the role of the January–March OMT in the southwestern Indian Ocean in predicting ISMR to those using other oceanic indices, which are defined by January–March mean SSTs and area-averaged regions where NINO3.4 (El Niño), El Niño Southern Oscillation Modoki Index (EMI), and Indian Ocean Dipole Mode Index (DMI) are conventionally defined (the EMI as a coupled ocean-atmosphere phenomenon in the tropical Pacific Ocean differs from El Niño Southern Oscillation in its spatial and temporal characteristics^18–20^. A recent study showed that teleconnections associated with EMI also influence the rainfall over India and South Africa^21^). The present study is useful because although there is typically modest variability in the monsoon season rainfall, small variations are very important for the agriculture and the economy of a country.

Since the Indian Ocean seasonal and intraseasonal variations are coupled^22^, the OHC and OMT, which depend on D_26_ in this case, have both spatial and temporal variations in this region. The D_26_ varies from 10 m to 100 m (Fig. 1a) and the OHC varies from 0 to 100 kJ cm^−2^(Fig. 1b), depending on the mixed layer processes and the net heat gain at the surface. January–March, a transition period between the winter and the southwest monsoon seasons in which the winds and currents in the tropical Indian Ocean change their patterns, has a better predictive value as shown in the results section of this paper. The surface temperature during this season in the Indian Ocean south of 20°S is less than 26 °C, meaning that D_26_ is undefined. Hence, OHC estimated with respect to the D_26_ reference depth is also undefined or arbitrarily set to zero. Both D_26_ and OHC during January–March are moderate in the southwestern Indian Ocean (the rectangular box in Fig. 1(a)), which is the region of interest in the present study. The January–March climatic mean D_26_ is 59 m and the corresponding OHC is 50 kJ cm^−2^ in this region. The Pearson’s correlation coefficient between OHC and D_26_ in this region is ~0.9, illustrating the strong control D_26_ exerts on OHC.

In this paper, we use this region and compare the OMT performance in relation to the other oceanic indices mentioned earlier, but focus on the sign of the monsoon rainfall anomaly in terms of the sign of the Percentage Deviation (PD), the difference in rainfall with respect to the mean value, expressed as a percentage, without finding quantitative relation with rainfall anomaly. Nanjundiah *et al*.^23^ also assessed the ability of seven atmospheric-ocean models to predict the sign of the ISMR anomaly. Other oceanic parameters such as ODMI, El Niño or EMI also provide a qualitative prediction^18,24,25^.

## Data and Approach

We analyzed 25 years (1993–2017) of monthly rainfall data provided by the India Meteorological Department (www.imd.gov.in/section/nhac/dynamic/data.htm). This rainfall time series used 6955 rain gauges spread over India, including the hilly regions^26,27^ (Fig. S1). The delayed time data of OHC and D_26_ spanning 30°S to 30°N and 40°E to 100°E, with a grid spacing of 0.25° × 0.25°, is taken from the National Oceanic and Atmospheric Administration, Atlantic Oceanographic and Meteorological Laboratory (ftp.aoml.noaa.gov). The OHC field, the available heat energy in the upper layer, has dimensional inequality with SST and cannot be used in place of SST in the numerical models. To overcome this limitation, we converted the OHC fields to OMT fields using a few assumptions^17^. As the OHC from satellite and *in situ* derived observations has a good correlation having regression slope of close to one and a y-intercept of almost zero^28^, we used the following equation to compute OMT from OHC and D_26_ following Ali *et al*.^17^.

1 $$OHC=\rho {C}_{p}{\int }_{0}^{{D}_{26}}(T-26)dz$$

where, ρ is the density of the seawater, C_p_ the specific heat capacity at constant pressure, T, the temperature (°C) of each layer of dz thickness, and D_26_, the depth of 26 °C isotherm. If we assume a mean temperature of the layer (from the surface to D_26_) as OMT, the above equation can be simplified as:

2 $$OHC=\rho {C}_{p}(OMT-{\rm{26}}){D}_{{\rm{26}}}$$

From the above equation, OMT can be written as:

3 $$OMT=(\frac{OHC}{\rho {C}_{p}{D}_{{\rm{26}}}})+{\rm{26}}$$

Since OMT and SST have the same units (unlike SST and OHC), the two parameters have the potential to be easily compared and replaced. OMT values thus obtained have data gaps at a few locations at 0.25 degrees spatial resolution. Hence, the monthly averaged OMT values have been obtained with a grid spacing of 0.5° × 0.5° using Cressman’s^29^ technique.

The best and most reliable approach involves calculating OHC or OMT from *in situ* temperature profiles. Since the temporal and spatial coverage of *in situ* observations is limited, we used satellite estimations providing more coverage in space and time. Nagamani *et al*.^28^ compared the satellite-derived OHC with the estimations from *in situ* hydrographic observations and reported a root-mean-square difference of 20.95 kJ cm^−2^, with a coefficient of determination, R^2^, of 0.65 and a bias of 11.27 kJ cm^−2^. Since we are using OMT in this study, we then further compared the OMT estimated from equation (3) with that estimated from *in situ* hydrographic observations. The statistical comparison (Fig. S2 and Table S1) between the OMT estimated from *in situ* measurements (obtained from www.nodc.noaa.gov) and that estimated from the satellite-derived OHC has a scatter index, defined as root mean square error normalized to the *in situ* data mean, of 0.02 and coefficient of determination (R^2^) of >0.77 for both the North Indian Ocean, as a whole, and for the southwestern Indian Ocean, during January–March. Other statistical parameters for this comparison are given in Table S1.

We used monthly SST data from the Met Office Hadley Centre’s sea ice and SST data set^30^ with a grid spacing of 1° × 1°, to compute the PD of January–March mean SST in the southwestern Indian Ocean. To show the importance of OMT compared to other oceanic indices, such as NINO3.4, EMI, and the DMI, these indices are obtained from the respective organizations as discussed below. Hadley monthly SST data^30^ was used to compute these parameters. We do not compute these indices separately; instead, we use them as they are for our comparison. The monthly NINO3.4 index, which is a central tropical Pacific SST based-index representing different phases of El Niño, is from Royal Netherlands Meteorological Institute climate explorer. The monthly EMI and DMI are from Japan Agency for Marine-Earth Science and Technology. DMI, representing the IOD phases and intensities during its life cycle, is defined as the gradient in SST anomaly between the western equatorial Indian Ocean (50°E–70°E and 10°S–10°N) and the southeastern equatorial Indian Ocean^2,3^ (90°E–110°E and 10°S–0°N). In addition, we computed another new index called ODMI, which is similar to DMI but using OMT in place of SST (the area average OMT anomaly gradient between the western equatorial Indian Ocean (50°E–70°E and 10°S–10°N) and the southeastern equatorial Indian Ocean^2,3^ (90°E–110°E and 10°S–0°N). OHC obtained from satellite observations is up to the depth of 26 °C isotherm. To check if a shallower or a deeper layer would have been a better choice, we used Hadley temperature and salinity profiles (version: EN4) data^31^ to compute OHC of a few more depths. Complete details and description of the data/indices used in the present study can be found in Table 1.

**Table 1:** Details of the data and variables used in the present study.

Parameter/Index	Variable used in computing	Grid spacing	Data Source	Study Region	Reference
Indian Summer Monsoon Rainfall (ISMR)	Station Rainfall Data	0.25° × 0.25°	1	Indian mainland (Fig. S1)	^26, 27^
Ocean Mean Temperature Percent Deviation (OMT PD)	OHC, D_26_	0.25° × 0.25°	2	(50°E–70°E, 10°S–0°N)	^17^
Sea Surface Temperature Percent Deviation (SST PD)	Sea Surface Temperature	1° × 1°	3	(50°E–70°E, 10°S–0°N)	^30^
Dipole Mode Index from Sea Surface Temperature (DMI)^a^	Sea Surface Temperature	1° × 1°	4	(50°E–70°E, 10°–10°N); (90°E–110°E, 10°S–0°N)	^2, 30^
Dipole Mode Index from Ocean Mean Temperature (ODMI)	OHC, D_26_	0.25° × 0.25°	2	(50°E–70°E, 10°S–10°N); (90°E–110°E,10°S–0°N)	^2, 17^
El Nino/Southern Oscillation (NINO3.4)^b^	Sea Surface Temperature	1° × 1°	5	(5°N–5°S, 170°W–120°W)	^30^
El Nino Modoki Index (EMI)^c^	Sea Surface Temperature	1° × 1°	4	(165°E–140°W, 10°S–10°N); (110°W–70°W, 15°S–5°N); (125°E–145°E, 10°S–20°N)	^20, 30^
Hadley profile data (Version: En4)	Temperature & Salinity profiles	1° × 1°	3	(50°E–70°E, 10°S–0°N)	^31^
*In situ* OMT data	Temperature & Salinity profiles	—	6	(30°E–100°E, 30°S–30^o^N)	^36^

The time period for all the datasets is 1993–2017.

Date Source references: 1-India Meteorological Department; 2-National Oceanic and Atmospheric Administration Atlantic Oceanographic and Meteorological Laboratory; 3- Met Office Hadley Center; 4-Japan Agency for Marine-Earth Science and Technology 5-Royal Netherlands Meteorological Institute climate explorer and 6-National Oceanic Data Center, National Oceanic and Atmospheric Administration.

^a,b,c^These parameters are directly obtained from respective centres, which used Hadley SST for the computations.

For an easy comparison between OMT, SST, and ISMR, the PDs of these three parameters are calculated. For example, the ISMR PD is calculated as the ratio of the deviation of a given year seasonal mean value from its June–September climatological mean value, divided by the climatological mean, and then multiplied by 100 as shown below:

4 $${\rm{ISMR}}\,{\rm{PD}}=({\rm{R}}-{\rm{R}}\_{\rm{clim}})/{\rm{R}}\_{\rm{clim}}\,\ast \,100$$

Here, R is the June–September ISMR in a given year and R_clim is June–September climatological mean of ISMR (887.5 mm in this case). Similarly, we computed OMT and SST PDs for different months from January to May and for different 3-month combinations starting from January–March to March–May. Then, the monthly DMI, ODMI, NINO3.4, and EMI indices are used to compute the January–March average values.

## Results

### OMT and SST variabilities

**Figure 2:**
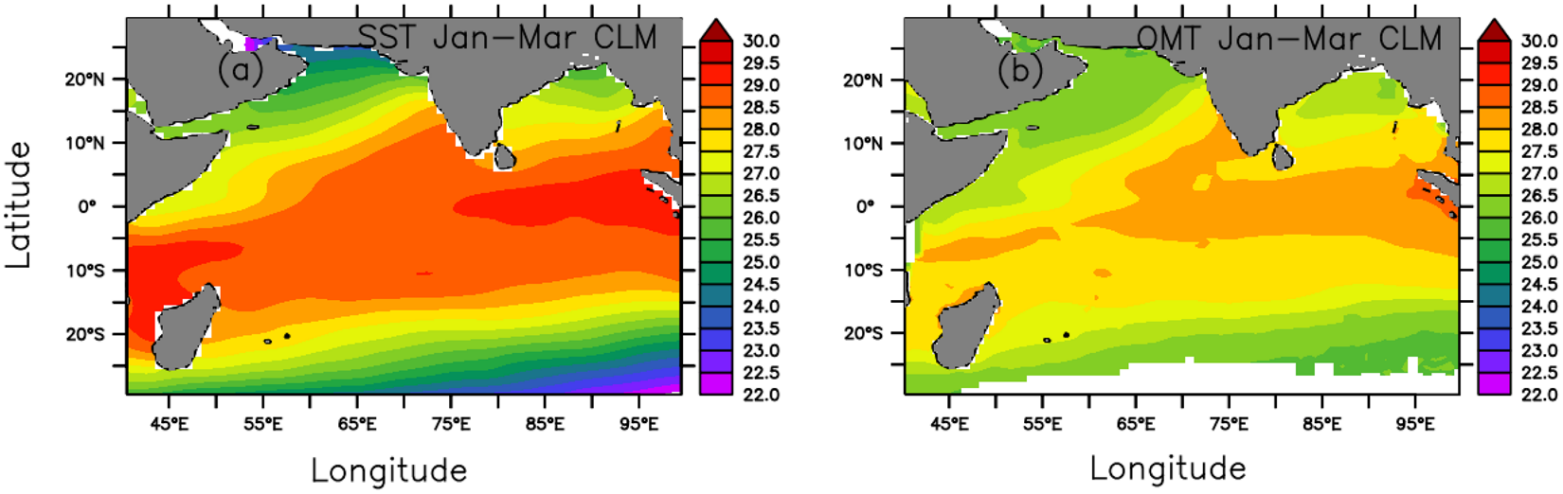
Spatial distribution of January–March mean values of (**a**) SST and (**b**) OMT in the Indian Ocean averaged over the period 1993–2017. This figure was generated using PyFerret V7.1.

Spatial distributions of January–March climatic mean SST and OMT in the Indian Ocean during 1993–2017 are shown in Fig. 2(a,b), respectively. Since OMT is an averaged temperature of a layer, it is, therefore, less than SST in the entire region. Hence, for a better comparison, we computed the coefficient of variation, defined as the relative magnitude of the standard deviation to the average value, for these two parameters. The coefficient of variability of OMT (Fig. S3 (a)) is less than that of SST (Fig. S3 (b)) almost in the entire region. This is evident because SST is affected by atmospheric parameters, such as winds and clouds/radiation, and is, therefore, more variable than OMT. The average coefficient of variation for monthly SST (0.02) for January–March during 1993–2017 (75 values at each grid point) is double that for OMT (0.01) both for the North Indian Ocean, as a whole, and for the southwestern Indian Ocean. On the contrary, these values remain same for both SST and OMT if January–March seasonal averages (25 values at each grid point) are used in computing the coefficient of variation, as the shorter timescale variations average out. Thus, SST variations that have shorter timescales compared to those of OMT introduce more noise and cause lower correlations with ISMR. Hence, OMT variations, which are more stable and consistent with a lower spread, have a better predictability compared to SST, having more noise.

### Comparison of ISMR with OMT and SST PDs

**Figure 3:**
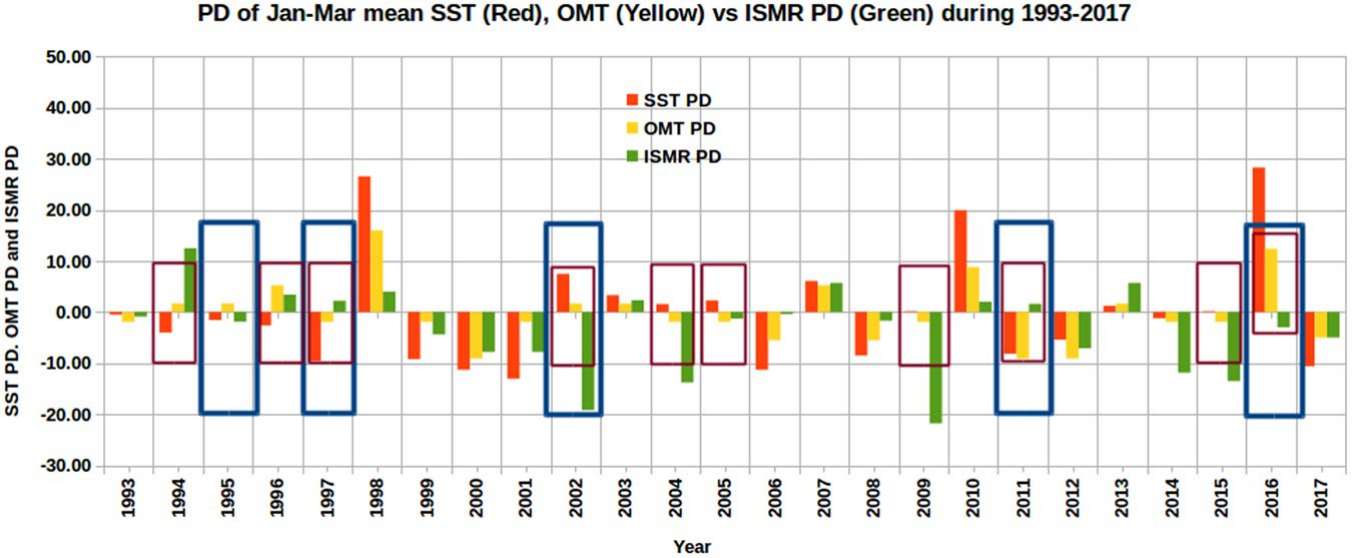
ISMR PD compared with January–March mean SST and OMT PDs in the Southwestern Indian Ocean region during 1993–2017. Blue (red) rectangle boxes in the figure denote the mismatching years between ISMR PD and OMT PD (SST PD). This figure was produced using Libre Office Version: 5.1.6.2.

We obtained the correlation coefficient for ISMR PD versus SST and OMT PDs separately for different months from January to May and for different 3-month combinations starting from January–March to March-May (Table S2). OMT PD correlates better with ISMR PD compared to SST PD for the three seasons and five months. Similarly, we compared the number of mismatching years if ISMR is predicted using SST and OMT PDs for the above months and 3-month seasons (Table S3). The number of mismatching years is least (5 out of 24) if January–March OMT PD is used. From these two analyses, we conclude that January–March OMT PD would be a better indicator of ISMR. As interannual anomalies in OHC near the equatorial Pacific lead those in equatorial SST by several months^8^, similar phase difference may also be present in the Indian Ocean. We further analyzed SST and OMT PDs in this study region to understand the predictability of this parameter for ISMR. For the purpose of easy visual comparison with ISMR PD, both SST and OMT PDs are multiplied by 10. A comparison of SST PD (red) and OMT PD (yellow) in the southwestern Indian Ocean with ISMR PD (green), shown in Fig. 3, indicates that the OMT PD has better predictability of the sign of the ISMR anomaly than SST PD. The OMT PD failed to predict the positive/negative ISMR (more/less than 887.5 mm) in only five years (1995, 1997, 2002, 2011, and 2016) out of the 25 years, marked as blue rectangles in Fig. 3. Except in 1995, the SST PD criterion also failed whenever OMT PD criterion failed, besides failures in other years. There are altogether 10 failures using SST PD (1994, 1996, 1997, 2002, 2004, 2005, 2009, 2011, 2015, and 2016); these are marked as red rectangles in Fig. 3. Thus, the success rate to predict whether ISMR is above- or below-average for OMT PD is 80% (20/25), whereas the success rate for SST PD is 60% (15/25). However, the magnitude of ISMR is not well predicted by either SST or OMT in a few years. Since the aim of this paper is to forecast the sign of ISMR, OMT will be shown to be better than SST for this goal. The ISMR PD is the greatest (12.50%) in 1994, followed by 2007 and 2013, each with 5.70%. The fact that only nine of 25 years have ISMR that is greater than the mean value (i.e., 887.5 mm) indicates that the overall performance (strength) of the monsoon has been declining. Nagamani *et al*.^16^ also reported an overall decreasing trend in ISMR, which is attributed to the increasing OHC of the Arabian Sea Mini Warm Pool region. Furthermore, the lowest negative deviations in ISMR are −21.80% and −19.20% during 2009 and 2002, respectively, whose magnitudes are much larger than the highest positive deviations of 12.50% in 1994 and 5.7% in both 2007 and 2014. This indicates that the rainfall departures from the climatological mean during the years with below-average values of rainfall are greater overall than rainfall departures during years with above-average values. This is critically important, particularly for water resources development in the region, because the deficit rainfall in below-average years is not compensated by excess rainfall in above-average years even without consideration of the flood water going to the oceans. During the moderate below-average years (e.g., 2004, 2015 and 2014), the ISMR PDs are −13.80%, −13.52% and −11.87%, respectively. These values are still more prominent than those during the above-average rainfall years. The maximum difference in ISMR PD between above- and below-average years is 34.30%, implying that the ISMR has high inter-annual variability, although normally the absolute value of this PD is much smaller than this extreme.

There are 16 (9) below- (above-) average rainfall years (green rainfall bars having negative (positive) values in Fig. 3) during the 25-year study period. The January–March OMT PD successfully predicts the ISMR for 13 out of 16 below-average rainfall years (1993, 1999, 2000, 2001, 2004, 2005, 2006, 2008, 2009, 2012, 2014, 2015, and 2017) but fails for three years (1995, 2002 and 2016). Out of these three below-average rainfall years, the ISMRs during 1995 and 2016 are influenced by IOD and El Niño Southern Oscillation; during 2002, the lower atmosphere over western India had an anomalous circulation^32^. Similarly, the OMT PD is successful in predicting the ISMR for 7 out of the 9 above-average rainfall years (Fig. 3 and Table 2) but fails for 2 years (1997 and 2011). By contrast, the SST PD successfully predicts 10 out of the 16 below-average rainfall years and 5 out of 9 above-average rainfall years (Fig. 3 and Table 2). Therefore, OMT PD is a better parameter than SST PD in predicting ISMR during our analysis period (1993–2017). These results are summarized in Table 2. However, modelling efforts considering other phenomena such as IOD and El Niño Southern Oscillation are required to improve the predictability of the ISMR.

**Table 2:** The success and failure years predicted using OMT PD and SST PD for 9 (16) above (below) average rainfall years.

Parameter	Above average rainfall years (9)			Below average rainfall years (16)		
	Success	Failure	Rate of success	Success	Failure	Rate of success
OMT PD	1994,1996,1998,2003,2007,2010, 2013	1997, 2011	78%	1993,1999,2000,2001, 2004,2005,2006,2008, 2009, 2012,2014, 2015, 2017	1995,2002, 2016	81%
SST PD	1998,2003,2007,2010, 2013	1994,1996,1997, 2011	55%	1993,1995,1999,2000, 2001,2006,2008, 2012,2014, 2017	2002,2004,2005,2009,2015, 2016	63%

### Statistical analyses

We carried out the following statistical analyses to assess whether OMT PD is a better ISMR predictor than SST PD:

#### Significance test

A 1000-sample permutations test revealed that the success rate for OMT PD is significant at the 1% level and that for SST PD it is significant at the 10% level. The difference between the success rates is significant at the 10% level.

#### The equitable threat score analysis

The equitable threat score accounts for the likelihood of random successes, with values > 0 indicating some skill, and a score of 1 indicating a perfect skill. We used separate equitable threat score to examine the effectiveness of determining the positive and negative signs. For SST, the equitable threat score is 0.020 for positive anomalies, and 0.094 for negative anomalies. Thus, SST has a little skill, but not much. In contrast, OMT has an equitable threat score of 0.295 and 0.405 respectively for positive and negative anomalies respectively. These differences are in part due to the probability of a false detection for an OMT-based estimate being about half that of an SST-based estimate.

#### Confounder analysis

To test whether OMT PD is a confounder (A confounding variable is one that may affect the dependent variable, other than the independent variable already in consideration) or not, first the coefficient of SST PD with ISMR is obtained in a linear regression (ISMR = a0 + b1* SST PD). Then OMT PD is added to the regression (ISMR = a0 + b1* SST PD + b2* OMT PD). The coefficient of SST (b1) has significantly changed by adding OMT PD, indicating that OMT PD is a confounder.

#### Comparison of standardized regression coefficients

A standardized beta coefficient compares the strength of the effect of each individual independent variable to the dependent variable. The higher the absolute value of the beta coefficient, the stronger the effect. Standardized beta coefficients are the coefficients we get if the variables in the regression were all converted to z-scores before running the analysis. The multiple regression analysis of ISMR with OMT and SST PDs has the standardized regression coefficients of 0.92 (significant at 3% level for OMT PD and −0.71 (significant at 8% level) for SST PD. The student T-test T values for these two parameters are 2.43 and −1.86 respectively. These two results show that OMT is better correlated to ISMR than SST.

### Comparison with other indices

**Figure 4:**
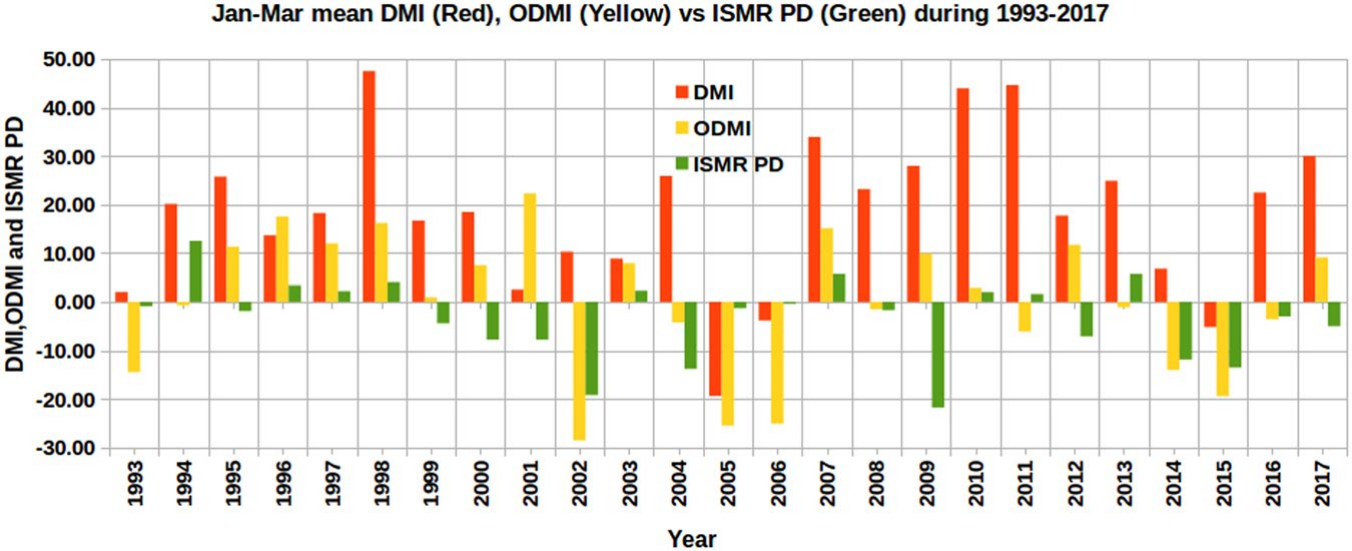
ISMR PD compared with January–March mean indices of DMI and ODMI during 1993–2017. Both DMI and ODMI are multiplied by 100 for easier visualization. This figure was produced using Libre Office Version: 5.1.6.2.

**Figure 5:**
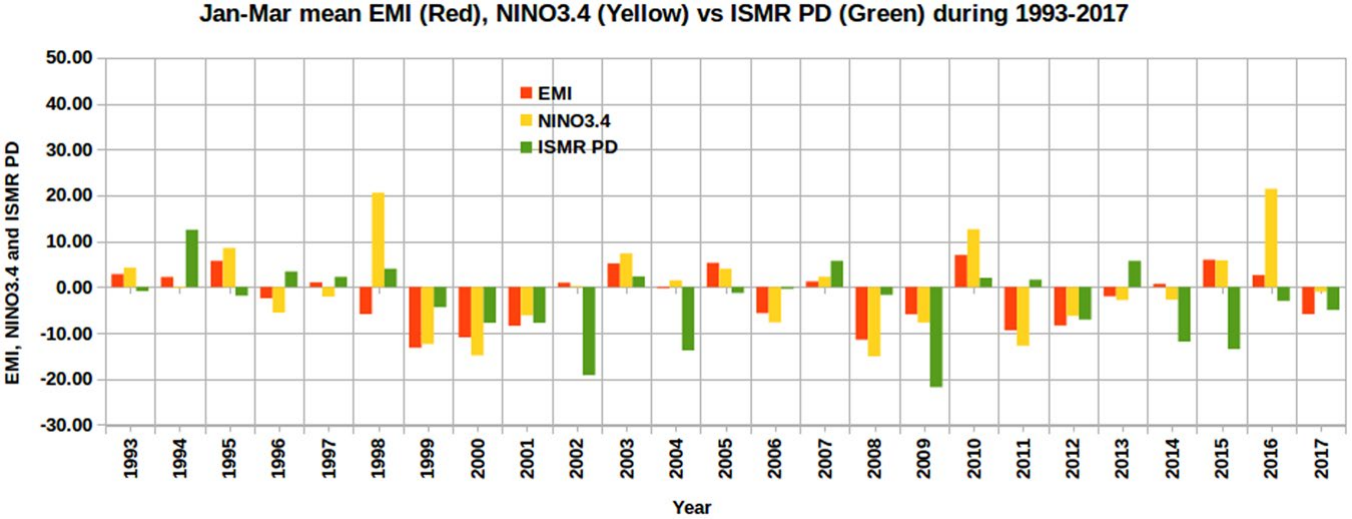
ISMR PD compared with January–March mean indices of EMI and NINO3.4 during 1993–2017. For a better comparison, both EMI and NINO3.4 indices are multiplied by 10. This figure was produced using Libre Office Version: 5.1.6.2.

In addition, the DMI and ODMI were compared with ISMR PD in Fig. 4. If we consider the annual average, 1996 and 2005 have negative DMI values, whereas the January–March average has negative values for 2005, 2006, and 2015. It is shown that the signs of DMI and ISMR do not match for 13 years, with a success rate of 48% (12/25). The success rate for ODMI is 60% (15/25) with 10 mismatching years. Similarly, we compared January-March mean EMI (red) and NINO3.4 index (yellow) with ISMR PD (green) in Fig. 5 and found that EMI and NINO3.4 have 11 mismatching years (i.e., a success rate of 14/25, or 56%) and 12 mismatching years (i.e., a success rate of 13/25, or 52%), respectively. Thus, of the six indices compared, OMT PD is better at predicting the sign of ISMR, with a success rate of 80%. The success rate of other indices varies between 48% and 60% (Table 3). The number of mismatches with OMT PD (5) is almost 50% less than other parameters (10–13). We obtain the January–March average values of DMI, ODMI, NINO3.4, and EMI monthly indices for the purpose of comparing January–March OMT PD, which has better predictability compared to SST PD (Tables S2 and S3). However, we also compared these monthly indices with SST and OMT PDs (Table S4). Except for ODMI (again an index computed with OMT) in January, OMT PD from January to March outperformed other indices even on monthly basis. This highlights that the OMT in the southwestern Indian Ocean during January–March is a key parameter for improved prediction of ISMR.

**Table 3:** Number of years sign of ISMR PD mismatching with OMT PD, SST PD, ODMI, DMI, NINO3.4 and EMI.

Parameter	No. of years with positive/negative signs [ISMR PD (9/16)]	Years mismatched with ISMR PD	No. of years mismatched (Success rate)
OMT PD	10/15	1995, 1997, 2002, 2011,2016	05 (80%)
SST PD	11/14	1994, 1996, 1997, 2002, 2004,2005, 2009, 2011, 2015,2016	10 (60%)
ODMI	13/12	1994, 1995, 1999, 2000,2001, 2009, 2011, 2012, 2013,2017	10 (60%)
DMI	22/03	1993,1995, 1999, 2000,2001,2002,2004, 2008,2009, 2012, 2014, 2016,2017	13 (48%)
NINO3.4	11/14	1993, 1994,1995, 1996, 1997, 2002,2004, 2005, 2011, 2013, 2015, 2016	12 (52%)
EMI	12/13	1993, 1995, 1996, 1998, 2002, 2005, 2011, 2013, 2014, 2015, 2016	11 (56%)

## Discussions

Among the six parameters studied, OMT PD averaged over January–March, performed better with two months lead time for a prediction. This statement is strongly supported by the differences in equitable threat scores, a 1000-sample permutation test, confounder analysis and comparison of standard regression coefficients indicating much more skill in the OMT-based forecast. Hence, OMT could be a better parameter as compared to SST, DMI, ODMI or EMI. This may be due to the fact that OMT better represents the upper ocean thermal energy conditions whose variations are mainly responsible for ISMR activity, whereas SST accounts only for the temperature of a very thin layer influenced by meteorological factors such as strong winds, evaporation, or thick clouds. In addition, compared to SST, OMT variations are more stable and consistent with less spread. Rajeevan and McPhaden^33^ reported that the relationship between warm water volume in the tropical Pacific and El Niño Southern Oscillation has a much stronger lead time than the El Niño Southern Oscillation-SST relationship in predicting ISMR. Lopez *et al*.^34^ related the variability of the heat content/transport in the South Atlantic ocean and variability of monsoon at global scales. Zhu *et al*.^35^ studied the seasonality between the thermocline depth and SST relationship in the eastern equatorial Pacific. They observed that the thermocline-SST relationship is clearly weaker in spring (with deeper thermocline) than that in other seasons. The cause, according to them, is even though OHC (or the thermocline depth) has the strongest persistence during spring, its variations may not be communicated to the surface (represented by SST). Thus, the link between OHC and SST is depth dependent. This, probably, would not have been the case if OMT were considered.

An important point worth discussing is the use of the mean temperature of the ocean layer down to the depth of 26 °C isotherm (the depth used for OHC computations). Although the use of OMT of this layer makes sense for TC studies as TC-induced mixing and cooling are weaker for higher values of OMT or OHC, such a reduced cooling during the pre-monsoon season cannot be ruled out. It could be related to vertical mixing. When OMT is high, mixing during the winter and spring does not cool SST as much, leading to warmer southwestern Indian Ocean waters, more moisture in the atmosphere, more moisture advected to the continent, and higher monsoon rainfall. Furthermore, there could be a remote oceanic influence associated with changes in OMT that SST does not capture. Westward-propagating Rossby waves could appear in OMT but not in SST, and then affect SST at the western boundary. The ocean thermal energy stored in the upper ocean represented by OMT in the southwestern Indian Ocean in January–March is generally a key parameter for the genesis and development of the following southwest monsoons. The variations of the stored ocean energy in different January–March may control the strength of the following ISMR by providing energy to the overlying atmosphere. This induces anomalous atmospheric circulation and moisture availability in the western Indian Ocean, including the Arabian Sea, a region where wind strength, direction, and moisture availability can significantly control the strength of ISMR. Thus, the use of SST is likely to provide poorer predictability of the ISMR relative to the use of OMT. These issues need to be addressed in a separate study, specifically making use of numerical models.

In this study, we compared only the positive/negative ISMR years with respect to the climatological mean of 887.5 mm. We did not categorize rainfall as the deficit, below normal, normal, above normal, and excess years as the India Meteorological Department does. We also did not attempt to quantitatively predict or compare the ISMR PD with OMT PD. The aim of the present paper is to show that OMT could be a better parameter than SST and other climate indices for monsoon prediction. One possible way to make quantitative predictions could be to replace SST with OMT in numerical weather prediction models, which, however, is beyond the scope of the present study.

To address if a much shallower or deeper layer than the depth of 26 °C would have been a better choice, we compute OMT PDs in southwestern Indian Ocean region for different layers using Hadley interpolated monthly mean temperature profiles with 1° spatial resolution. These temperature profiles are at 42 depth levels from 5 m to 5250 m. Out of the 25 years studied, the range of the annual mean D_26_ is 35–85 m with 21 (84%) observations varying between 40 m and 70 m. We computed OMT PDs at seven other depths of the surface (5 m) to 15.1 m, 25.2 m, 35.3 m, 45.4 m, 55.7 m, 100 m and 150 m. The number of years mismatching with ISMR PD varies from 11 (correlation: 0.11) with OMT PD of the surface to 100 m to 18 matching or seven years mismatching (correlation: 0.23) with OMT PD of the surface to 15.1 m. Thus, we conclude that using OHC down to D_26_ to compute OMT PD is a better choice, possibly because this depth varies depending upon the atmospheric factors.

Here, we demonstrated statistical evidence that OMT is a better predictive parameter for the sign of ISMR compared to SST, DMI, ODMI, EMI and Nino3.4. The success rate for OMT in predicting the sign of the ISMR anomaly is 80% and significant at greater than 1%, far better than the other parameters considered.

## Conclusion

Since the OHC, representing the mean temperature of the surface to D_26_ isotherm layer, has been recognized as an important parameter in cyclone forecasting, we extended the scope of this parameter to predict whether ISMR is above- or below-average value. We converted OHC to OMT so that the two parameters (SST and OMT) have the potential to be easily compared and replaced. Comparison of the correlation coefficient for ISMR PD versus SST and OMT PDs separately for different months from January to March and for 3-month averages from January-March to March-May revealed that OMT PD correlates better with ISMR PD compared to SST PD. Further, the number of mismatching years is least if OMT PD of January-March is considered. OMT PD is showing better performance than SST PD because of the following reasons: (1) The rapid heating or cooling events can quickly eradicate the thermal signatures of subsurface warm or cold features with which the atmosphere interacts rather than with the top thin layer of the ocean represented by SST alone and (2) SST variations that have smaller time scales introduce more noise and cause lower correlations with ISMR compared to OMT variations, which are more stable and consistent with lower spread. In addition, we also compared the OMT PD performance with ODMI another new index, which is similar to DMI but using OMT in place of SST, and other conventionally used climate indices such as NINO3.4, EMI, and DMI.

Equitable threat scores, confounder analysis, standardized regression analysis and a 1000-sample permutation test suggest that among the six parameters/indices used, the January–March mean OMT PD in the southwestern Indian Ocean has the best relationship with ISMR PD. Since OMT PD of January–March has a better predictive value, by the end of March, we expect to predict qualitatively whether the monsoon would be above or below its climatological mean (i.e., 887.5 mm). Similar to other indices or parameters such as DMI, or El Niño/NINO3.4 or EMI, OMT PD helps in a qualitative prediction of ISMR — whether the monsoon is above or below the long period average. Comparison of this parameter estimated for other depths revealed that OMT PD of D_26_ is a better choice, which could be because of the influence of the atmospheric factors on D_26_. The physical evidence for OMT in the southwestern Indian Ocean having a higher predictive value, and its ability to provide an improved quantitative prediction, need to be explored using numerical/dynamical models; here we present only the statistical evidence.

Although we have already provided some reasons why OMT failed to predict ISMR in some years, the failure of predicting ISMR using OMT in some years implies the complexity of monsoon system determining the vagaries of ISMR, which are not always controlled by the ocean energy alone represented by OMT in the southwestern Indian Ocean. For example, the influence from other ocean basins associated with ENSO, atmospheric processes related to Madden-Julian Oscillation, Tropical Intraseasonal Oscillation in the time scales of 10–20 days, and the impact from the extra-tropic regions, which are at least partly independent of OMT in Indian Ocean, may also be important contributors to the variability of ISMR in both time and space.

The OMT PD during January–March 2018 is −14.32, which may lead to below average rainfall during June–September 2018 with 80% probability.

## Electronic supplementary material

Supplementary Material

## Data Availability

OMT is estimated using the freely available data. While the other data are available in public domain, OMT data estimated by us can be shared via Dropbox.

## References

[1] Ashok K, Guan Z, Yamagata T (2001). Impact of the Indian Ocean dipole on the relationship between the Indian monsoon rainfall and ENSO. Geophysical Research Letters.

[2] Saji NH, Goswami BN, Vinayachandran PN, Yamagata T (1999). A dipole mode in the tropical Indian Ocean. Nature.

[3] Webster PJ, Moore AM, Loschnigg JP, Leben RR (1999). Coupled ocean-atmosphere dynamics in the Indian Ocean during 1997–98. Nature.

[4] Pickard, G. L. & Emery, W. J. Descriptive physical oceanography: an introduction. *Elsevier* (1990).

[5] Smith TM, Barnston AG, Ji M, Chelliah M (1995). The impact of Pacific Ocean subsurface data on operational prediction of tropical Pacific SST at the NCEP. Weather and Forecasting.

[6] Ji M, Leetmaa A (1997). Impact of data assimilation on ocean initialization and El Nino prediction. Monthly Weather Review.

[7] Latif M (1998). A review of the predictability and prediction of ENSO. Journal of Geophysical Research: Oceans.

[8] McPhaden, M. J. Tropical Pacific Ocean heat content variations and ENSO persistence barriers. *Geophysical research letters*, **30**(9) (2003).

[9] Meinen CS, McPhaden MJ (2000). Observations of warm water volume changes in the equatorial Pacific and their relationship to El Niño and La Niña. Journal of Climate.

[10] Xue Y, Leetmaa A, Ji M (2000). ENSO prediction with Markov models: The impact of sea level. Journal of Climate.

[11] Lin II, Goni GJ, Knaff JA, Forbes C, Ali MM (2013). Ocean heat content for tropical cyclone intensity forecasting and its impact on storm surge. Natural Hazards.

[12] Mainelli M, DeMaria M, Shay LK, Goni G (2008). Application of oceanic heat content estimation to operational forecasting of recent Atlantic category 5 hurricanes. Weather and Forecasting.

[13] Shay LK, Goni GJ, Black PG (2000). Effects of a warm oceanic feature on Hurricane Opal. Monthly Weather Review.

[14] Ali MM, Kashyap T, Nagamani PV (2013). Use of sea surface temperature for cyclone intensity prediction needs a relook. Eos, Transactions American Geophysical Union.

[15] Ali MM, Swain D, Kashyap T, McCreary JP, Nagamani PV (2013). Relationship between cyclone intensities and sea surface temperature in the tropical Indian Ocean. IEEE Geoscience and Remote Sensing Letters.

[16] Nagamani PV (2016). Heat content of the Arabian Sea Mini Warm Pool is increasing. Atmospheric Science Letters.

[17] Ali MM (2015). Relationship between ocean mean temperatures and Indian summer monsoon rainfall. Atmospheric Science Letters.

[18] Ashok, K., Behera, S. K., Rao, S. A., Weng, H. & Yamagata, T. El Niño Modoki and its possible teleconnection. *Journal of Geophysical Research: Oceans*, ***112*****(****C11****)** (2007).

[19] Weng H, Ashok K, Behera SK, Rao SA, Yamagata T (2007). Impacts of recent El Niño Modoki on dry/wet conditions in the Pacific rim during boreal summer. Climate Dynamics.

[20] Ashok K, Yamagata T (2009). Climate change: The El Niño with a difference. Nature.

[21] Ratnam, J. V., Behera, S. K., Masumoto, Y., Takahashi, K. & Yamagata, T. Pacific Ocean origin for the 2009 Indian summer monsoon failure. *Geophysical Research Letters*, ***37*****(****7****)** (2010).

[22] Schott, F. A., Xie, S. P. & McCreary, J. P. Indian Ocean circulation and climate variability. *Reviews of Geophysics*, ***47*****(****1****)** (2009).

[23] Nanjundiah, R. S., Francis, P. A., Ved, M. & Gadgil, S. Predicting the extremes of Indian summer monsoon rainfall with coupled ocean–atmosphere models. *Current Science*, 1380–1393 (2013).

[24] Ashok K, Guan Z, Saji NH, Yamagata T (2004). Individual and combined influences of ENSO and the Indian Ocean dipole on the Indian summer monsoon. Journal of Climate.

[25] Pokhrel S (2012). ENSO, IOD and Indian Summer Monsoon in NCEP climate forecast system. Climate Dynamics.

[26] Rajeevan, M., Bhate, J., Kale, J. D. & Lal, B. High resolution daily gridded rainfall data for the Indian region: Analysis of break and active monsoon spells. *Current Science*, 296–306 (2006).

[27] Pai DS (2014). Development of a new high spatial resolution (0.25 × 0.25) long period (1901–2010) daily gridded rainfall data set over India and its comparison with existing data sets over the region. Mausam.

[28] Nagamani PV (2012). Validation of satellite-derived tropical cyclone heat potential with *in situ* observations in the North Indian Ocean. Remote Sensing Letters.

[29] Cressman GP (1959). An operational objective analysis system. Monthly Weather Review.

[30] Rayner, N. A. *et al*. Global analyses of sea surface temperature, sea ice, and night marine air temperature since the late nineteenth century. *Journal of Geophysical Research: Atmospheres*, **108**(D14) (2003).

[31] Good SA, Martin MJ, Rayner NA (2013). EN4: Quality controlled ocean temperature and salinity profiles and monthly objective analyses with uncertainty estimates. Journal of Geophysical Research: Oceans.

[32] Bhat GS (2006). The Indian drought of 2002-a sub‐seasonal phenomenon?. Quarterly Journal of the Royal Meteorological Society.

[33] Rajeevan, M. & McPhaden, M. J. Tropical Pacific upper ocean heat content variations and Indian summer monsoon rainfall. *Geophysical Research Letters*, 31(18) (2004).

[34] Lopez H, Dong S, Lee S-K, Goni G (2016). Decadal Modulations of Interhemispheric Global Atmospheric Circulations and Monsoons by the South Atlantic Meridional Overturning Circulation. J. Clim..

[35] Zhu J, Kumar A, Bohua Huang B (2015). The relationship between thermocline depth and SST anomalies in the eastern equatorial Pacific: Seasonality and decadal variations. Geophysical Research Letters.

[36] NOAA National Centers for Environmental Information, National Oceanic and Atmospheric Administration (https://www.nodc.noaa.gov/OC5/woa13/woa13data.html).

