# Supplementary material for: Statistical Evidence for the Role of Southwestern Indian Ocean Heat Content in the Indian Summer Monsoon Rainfall
Source: Sci Rep. 2018 Aug 14;8:12092. doi: 10.1038/s41598-018-30552-0 (PMC6092415; doi:10.1038/s41598-018-30552-0)
Supplement: Supplementary file 1 — Supplementary Material [file 41598_2018_30552_MOESM1_ESM.docx]

**Statistical Evidence for the Role of Southwestern Indian Ocean Heat Content in the Indian Summer Monsoon Rainfall**

**Thandlam Venugopal^1,2^, M.M. Ali*^3,4^, M. A. Bourassa^3,5^, Y. Zheng^3^, G.J.Goni^6^, G.R. Foltz^6^ and M. Rajeevan**

**Author information & Affiliations**

^1^ Department of Physics, Novosibirsk State University, Novosibirsk, Russia-630090

^2^Department of Meteorology and Oceanography, Andhra University, Visakhapatnam, India

^3^ Center for Ocean-Atmospheric Prediction Studies (COAPS), Florida State University, USA.

^4^ Indian Institute of Tropical Meteorology, Pune, India

^5^Department of Earth, Ocean and Atmospheric Science, Florida State University, USA

^6^Physical Oceanography Division, Atlantic Oceanographic and Meteorological Laboratory (AOML)/NOAA, USA.

^7^Ministry of Earth Sciences, Government of India.

### **Corresponding author:**

MM Ali, mmali110@gmail.com

**Supplementary Figures and Tables**

**Figures:**


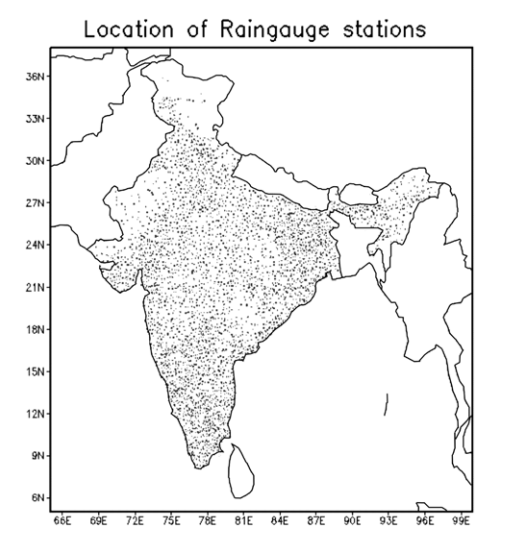


Figure S1: Distribution of rain gauge stations over the Indian subcontinent (Pai et al. 2014^1^, Courtesy : Editor MAUSAM – India Meteorological Department)


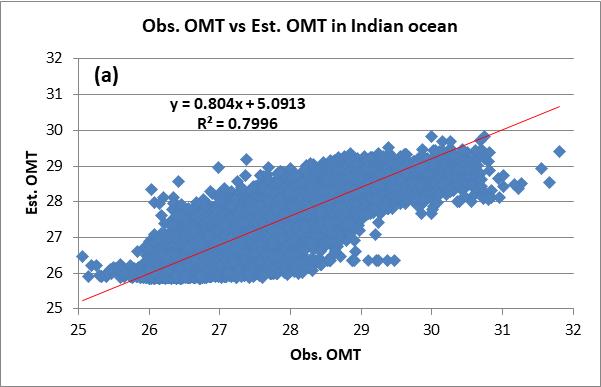

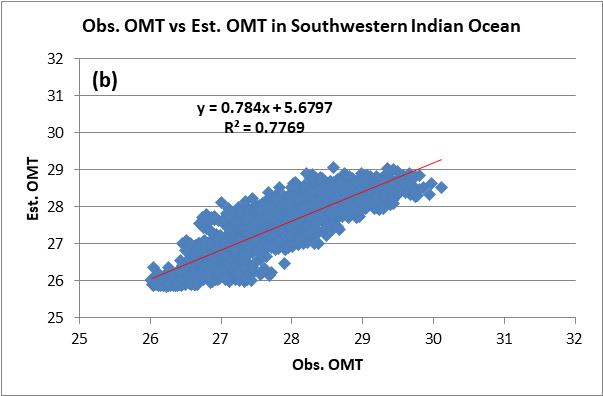


Figure S2: Scatter between the OMT estimated from the *in situ* profiles and that estimated from satellite derived OHC observations for (a) the north Indian Ocean and (b) the Southwestern Indian Ocean.


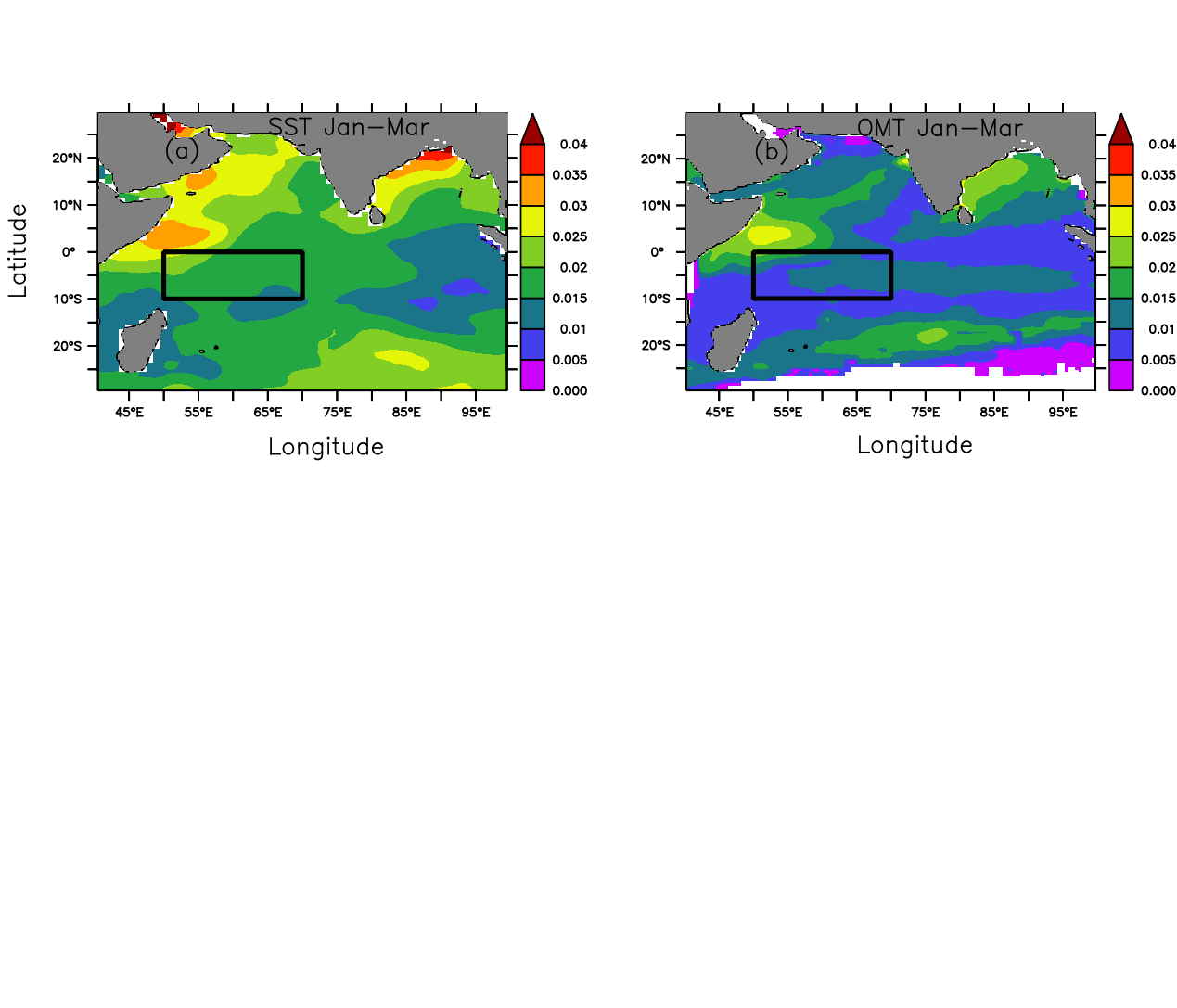


Figure S3: Coefficient of variability (relative magnitude of the standard deviation to the average value) of SST and OMT during 1993–2017.

**Tables:**

Table S1: Statistical comparison between the OMT estimated from the *in situ* profiles and estimated from satellite derived OHC observations for the north Indian Ocean and the Southwestern Indian Ocean.

| Region | No.of observations | RMSE | Correlation  coefficient | Bias | Mean | SI |
| --- | --- | --- | --- | --- | --- | --- |
| Indian Ocean | 71225 | 0.54 | 0.894 | -0.398 | Obs: 28  Est : 27.6 | 0.02 |
| Southwestern Indian Ocean | 5244 | 0.52 | 0.881 | -0.383 | Obs: 28.07  Est: 27.69 | 0.02 |

Table S2: Seasonal and Monthly correlation coefficients of ISMR PD with SST and OMT PDs

| Parameter/Month | January-March | February-April | March-May | January | February | March | April | May |
| --- | --- | --- | --- | --- | --- | --- | --- | --- |
| SST PD | 0.09 | 0.13 | 0.02 | 0.12 | 0.21 | 0.01 | 0.02 | 0.06 |
| OMT PD | 0.31 | 0.24 | 0.08 | 0.33 | 0.25 | 0.24 | 0.14 | 0.11 |

Table S3: No. of years sign of ISMR PD mismatching with SST & OMT PDs on monthly and 3-month average basis.

| Period | SST PD | OMT PD |
| --- | --- | --- |
| January | 9 | 11 |
| February | 9 | 9 |
| March | 9 | 7 |
| April | 12 | 10 |
| May | 14 | 14 |
| Jan-Mar | 9 | 5 |
| Feb-Apr | 12 | 6 |
| Mar-May | 11 | 13 |

Table S4: Monthly correlations of different indices with ISMR PD

| ISMR PD versus | | | | | | |
| --- | --- | --- | --- | --- | --- | --- |
|  | SST PD | OMT PD | DMI | ODMI | NINO 3.4 | EMI |
| Jan | 0.12 | 0.33 | 0.28 | 0.46 | 0.21 | 0.18 |
| Feb | 0.21 | 0.25 | 0.14 | 0.04 | 0.15 | 0.08 |
| Mar | 0.01 | 0.24 | 0.14 | 0.09 | 0.17 | 0.14 |

Reference:

1. Pai, D. S., Sridhar, L., Rajeevan, M., Sreejith, O. P., Satbhai, N. S. &Mukhopadhyay, B. Development of a new high spatial resolution (0.25×0.25) long period (1901–2010) daily gridded rainfall data set over India and its comparison with existing data sets over the region. *Mausam*, 65(1), 1-18 (2014).
